# Economic analysis of the different endodontic instrumentation techniques used in the Unified Health System

**DOI:** 10.1186/s12903-022-02369-x

**Published:** 2022-08-11

**Authors:** Laura Paredes Merchan, Livia Fernandes Probst, Ana Clara Correa Duarte Simões, Augusto Cesar Santos Raimundo, Yuri Wanderley Cavalcanti, Denise de Fátima Barros Cavalcante, João Victor Frazão Câmara, Antonio Carlos Pereira

**Affiliations:** 1 Department of Public Health, Piracicaba Dental School, Universidade Estadual de Campinas - UNICAMP, Piracicaba, Brazil; 2 Department of Community Dentistry, Piracicaba Dental School, Universidade Estadual de Campinas - UNICAMP, Piracicaba, SP Brazil; 3 Department of Clinical and Social Dentistry, Federal University of Paraíba, João Pessoa, PB Brazil; 4 Department of Public Health, Israelita Albert Einstein Hospital, São Paulo, Brazil; 5 Clinic of Operative Dentistry, Periodontology and Preventive Dentistry, Saarland University Hospital, Homburg, Saar Germany

**Keywords:** Endodontic treatments, Health evaluation, Oral health

## Abstract

**Background:**

To assess the financial impact of incorporating a new (reciprocal) technology into endodontic treatments in the public health system (SUS).

**Methods:**

This was a economic evaluation study (comparing the 3 different endodontic instrumentation techniques—manual, rotary and reciprocating), allocative efficiency analysis to optimize existing resources in the SUS, and financial contribution impact analysis of incorporation of a new technology. Thirty-one (31) 12 years-old volunteers were evaluated.

**Results:**

The incremental cost-effectiveness ratio (ICER) was calculated at R$1.34/min, − R$0.60/min and BRL 0.10/min for the single-rooted, bi-rooted and tri-rooted teeth, respectively, when the rotary technique was compared with the manual type. In turn, the ICER was R$ 21.04/min, − R$ 0.73/min and − R$ 2.81/min for the 3 types of teeth, respectively, when the reciprocating technique was compared with the manual type. The incremental financial impact of replacing manual endodontic with rotary endodontic treatments would be − R$ 2060963.66 in the case of single-rooted teeth, but the number of treatments would also be reduced (− 19,379). In the case of two-rooted teeth, the incremental financial impact would be BRL 34921540.62 with the possibility of performing an additional 204,110 treatments. In turn, BRL 11523561.50 represented the incremental financial impact for teeth with 3 or more roots and with an increase of 72,545 procedures. When we analyzed the incremental financial impact of replacing manual endodontic with reciprocating endodontic treatments, it would be − R$ 730227.80 in the case of single-rooted teeth, allowing for an additional 2538 treatments. In turn, R$ 21674853.00 represented the incremental financial impact for bi-radicular teeth, with an increase of 121,700 procedures. In the case of two-rooted teeth, the incremental financial impact would be BRL 13591742.90 with the possibility of performing an additional 40,670 treatments.

**Conclusions:**

The reciprocating technique could improve access to endodontic treatment in the SUS as it allowed a simultaneous reduction in clinical time and associated costs. However, the higher number of endodontic treatments performed would have a financial impact.

**Supplementary Information:**

The online version contains supplementary material available at 10.1186/s12903-022-02369-x.

## Background

Oral health comprises a condition in which the person is free from pain, discomfort and changes in the mouth and face. However, access to oral health represents a major and neglected challenge to the global population. For example, the worldwide prevalence of untreated dental caries in permanent teeth was estimated to be 29.4% in 2017 [1].

When caries progresses and leads to cavitation, the condition can cause considerable pain and discomfort, and when it involves the dental pulp, it can also cause infection with risk of sepsis and tooth loss [1]. With irreversible impairment of the dental pulp, it is necessary to perform endodontic treatment to inactivate and remove microorganisms and their toxic metabolites in order to eliminate inflammation [2].

The chemical–mechanical preparation stage that involves instrumentation, irrigation, flooding and aspiration of the root canal is one of the most important stages of this treatment and is susceptible to accidents and complications [3]. These challenges have led to a constant search for improvement in the quality of root canal preparation and has motivated the transition from manual instrumentation to automation in contemporary clinical practice. The development of new technologies for root canal instrumentation, such as rotary and reciprocating techniques, has resulted in increased costs associated with endodontic treatment.

The maintenance of teeth allows continuity in the performance of their function, positively affecting the health of the individual. On the other hand, as endodontic treatment is more complex than the extraction of damaged teeth, its large-scale implementation in public health requires not only a change of philosophy, but also of investment. Dental care is one of the biggest contributors to these health care costs; in fact, it is estimated that the management of dental disease generates costs of 357 billion dollars a year worldwide, where it is estimated that more than 15 million people receive endodontic treatment each year, and per day 41,000 teeth are endodontically treated [4, 5].

In the Unified Health System (SUS), endodontic treatment began to be regulated, financed and performed as from 2004, through Ordinance No. of Dental Specialties (DSC). This treatment is financed exclusively with public resources from taxes, including access to oral health care [6]. According to data from the Ministry of Health—SUS Outpatient Information System (SIA/SUS), in the last 5 years (December/2015 to December/2020) a total of 2,820,459 endodontic fillings and retreatments of permanent teeth with single-roots, with two and three or more roots were performed in Brazil [6].

Considering the high demand for endodontic treatment in Brazil and the need to seek more efficient interventions, even in scenarios with limited financial resources, it is opportune to conduct an economic evaluation to compare the three endodontic techniques used in SUS (manual, rotary and reciprocating). Public management in oral health depends on information about the costs and effectiveness of different treatments to make decisions on the selection of treatments to be made available to the population [7, 8]. The intervention that incurs the lowest cost and is the most effective will the type that would be most rational to implement if the objective is to make the system efficient [7, 8]. This makes economic evaluations an essential component for public health systems. In view of the foregoing, the objective of this study was to conduct a economic analysis (by comparing the three different endodontic instrumentation techniques—manual, rotary and reciprocating) and an allocative efficiency analysis to optimize the use of existing resources in the SUS, including investigation into the financial impact of incorporating a new technology.

## Methods

### Planning the economic analysis of health

This was an economic analysis based on a single study for which project was approved by an Ethics Committee for Research with Human Beings of the Campinas State University (CAAE 29931720.4.0000.5418). A term of Free and Informed consent was obtained from all the children’s patients and/or their legal guardian(s). This article was described in accordance with the Consolidated Health Economic Evaluation Reporting Standards (CHEERS) Statement [9]. All methods were carried out in accordance with relevant guidelines and regulations (declaration of Helsinki).

### Population and subgroups

This economic evaluation study considered the target population to be SUS users with indication for endodontic treatment in a permanent tooth. Thus, patients with permanent dentition, adults and adolescents from 12 years of age, referred to DSC II with indication for endodontic treatment of teeth with single, two, three or more roots were included in the research. Patients referred with indication for endodontic retreatment were excluded.

### Context and location

Data on the effectiveness of each instrumentation technique were obtained from a survey conducted at the DSC of a medium-sized city (400,000 inhabitants) in the interior of the State of São Paulo/Brazil.

### Study perspective

For this study, the perspective of the municipal manager of the Unified Health System (SUS) was adopted; he/she is responsible for paying the direct costs related to payment for endodontic treatment in DSCs.

### Comparators

The interventions compared were the three endodontic techniques used in SUS for instrumentation of root canals, namely, manual, rotary and reciprocating. The stages of endodontic techniques differ only in the sequence and use of specific files for each system, and single obturation cones in mechanized instrumentation. All techniques, in all groups of teeth, were performed in a single session due to the expertise of the professional trained in this specialty.

An important factor to note is that in accordance with the recommendation made by the SUS, patients referred to the DSC are previously treated in primary care, where a prior radiographic examination is performed as part of the diagnosis, followed by access to the pulp chamber and intracanal medication, thus initiating inactivation and removal of the microorganisms [6].

Initially, a pilot study with a small number of patients was conducted, for the sole purpose of establishing the costs of materials, instruments and equipment, in addition to measuring the average time taken to perform the techniques for uni, bi and tri-rooted teeth. This study was not designed to be a primary study and does not follow the rules of a clinical study; however, it is an economic evaluation.

In the conventional manual endodontic technique, the files used in the biomechanical preparation were of the Kerr type, selected according to the diameter and caliber of the root canals of uni, bi or tri-rooted teeth. Instrumentation was always interspersed with irrigation with chlorhexidine and saline solution and subsequent aspiration. Then, Gates Glidden burs were used in the cervical-apical direction, after determining the working length with the memory file. After thorough washing and drying with absorbent paper cones, the main gutta percha cone was selected; to finish the technique, root canal obturation was performed by insertion of main and secondary cones, followed by the thermoplasticized core carrier condensation technique that was finalized with provisional sealing.

The difference between automated techniques (rotary and reciprocating) lies in the specific files used in these systems. After using the hand files (a common instrument for the three techniques tested) in the canal instrumentation phase, a rotary file or reciprocating file with the caliber and diameter according to the anatomy of each tooth was used. The latter files were driven by a rotary motor, according to the instrumentation technique used by endodontists with extensive experience in the area.

The subsequent remaining stages of treatment were similar in the three techniques tested. Clinical procedures were performed by a single endodontist who not only had extensive experience in the public health system but also had training in the three techniques tested. From the list of patients referred to the DSC for endodontic treatment, 31 individuals were invited to participate in the research. They were approached in the waiting room before the dental consultation began.

Each technique was performed in a randomized manner, using the “Analysis Tool” of the Microsoft Excel computer program for randomization purposes. Of the 31 endodontic treatments, 11 were performed with the manual technique, 11 with the rotary technique and 9 with the reciprocating technique.

### Time and discount rate

The stipulated time horizon was 1 year. No discount rate was applied either to costs or effectiveness, as the time horizon did not include a minimum of 3 years of analysis [10].

### Health outcome and effectiveness measurement

The effectiveness considered in this study was the measure of time taken to perform instrumentation of the root canals, stipulated as minutes and seconds based on the results of the primary research carried out at the DSC. The instrumentation time was chosen as the measure of effectiveness due to its importance to the public health service.

The timer was set to start at the beginning of root canal instrumentation. Prior to the clinical procedures, the volunteer was welcomed by the endodontist, who performed local anesthesia, when necessary, and removed the provisional sealing material since these patients had previously been examined at the primary health units and were duly referred to the DSCs.

For all treatments, the working time count began at the beginning of the root canal instrumentation and ended when the absolute isolation was removed. The techniques were standardized by a professional specialist in endodontic treatments who worked at the DSC II (Dental Specialty Center) in the city of Piracicaba, where data collection was carried out.

### Resource and cost estimation

The bottom up (micro costing) approach to estimation of resources and costs, with calculation of the direct costs was adopted, and involved three steps [7]:

1. Identification of categories of resources relevant to the assessment;
2. Measurement of the quantities of resources used, in physical units; and
3. Valuation of consumed resources in monetary terms.

The resources used were thus identified and quantified according to their use in the treatment, and monetary values were assigned to them. Since the perspective of the study was that of the municipal manager, the cost obtained from the website Banco de Preços em Saúde (BPS) (http://portalms.saude.gov.br/gestao-do-sus/economia-da-saude/banco-de-precos-em-saude) was considered for the material. In addition to searches for items not found in the BPS were made in manuals and on sites for sale of medical/dental materials.

Some assumptions were adopted during the collection of all values referring to items of material/instruments, such as: the calculation was justified by the different periods of consumption of the items on the list. This included disposable items, calculated by the number of units used and other items of a permanent nature (manufacturer indicates 5 years which amounts to 1265 working days), for which it is necessary to dilute the value by period such as the number of consultations performed (this parameter was obtained according to the average of 8 consultations/patients seen daily at the DSC). For example, in the case of rotary instrumentation equipment/engine, we initially searched for the cost of this item on sales sites, then diluted the period by the 1265 working days (to obtain the R$/day value, and then divided by 8 (number of patients seen at the DSC daily) to obtain the R$ of each use of this item of equipment per patient.

For costs related to human resources, the clinical hours of work of an endodontist and an oral health assistant (ASB) hired in the public health sector were considered. The State of São Paulo was used as a reference for calculating the average costs of human resources. Regarding Human Resources costs, the salaries used to obtain the average were also exempt from additions such as 13th salary, bonus and/or vacation pay.

### Currency, price date and conversion

Monetary values were presented in Brazilian reais (R$).

#### Methods of calculating the incremental cost-effectiveness ratio and the allocative efficiency of financial resources

The incremental cost-effectiveness ratio (ICER) was calculated by the difference in costs between the technologies divided by the difference in effectiveness, according to the formula expressed below:

$${\text{RCEI}} = \frac{{{\text{Cost}}_{{{\text{alternative}}}} - {\text{Cost}}_{{{\text{tradicional}}}} }}{{{\text{Effectiveness}}_{{{\text{alternative}}}} - {\text{Effectiveness}}_{{{\text{tradicional}}}} }}$$

Therefore, two ICER values were calculated. The ICER of the rotational technique (alternative 1) in comparison with the manual technique (traditional) and the ICER of the reciprocating technique (alternative 2) in comparison with the manual technique (traditional).

To calculate the allocative efficiency and the need for total investment to increase the diffusion of endodontic treatment in the SUS, we combined estimates of population participation and treatment costs. The eligible population in this case was calculated using the measured demand method, which is considered more appropriate for aiding decision-making [9]. For this purpose, the outpatient production found in TABNET/DATASUS was considered. In 2019, the reference year, 616,487 endodontic treatments were registered and approved in Brazil in 2019, as shown in Table 1.

**Table 1:** Number of endodontic treatments performed in 1 year according to each technique

Procedure	Quantity according to technique		
	Manual^a^	Rotary^b^	Reciprocal^b^
Endodontic treatment of single-rooted permanent tooth	218.141	198.762	220.679
Endodontic treatment of biradicular permanent tooth	178.676	382.786	300.376
Endodontic treatment of a permanent tooth with three or more roots	219.670	292.215	260.340

^a^Source: Number of endodontic treatmentss performed in 2019 according to Datasus/Tabnet. Ministry of Health—SUS Outpatient Information System (SIA/SUS)

^b^Calculated from effectiveness data

This meant that in the country, this was the demand and also the installed capacity to meet it. From these data, we assumed that in the reference scenario, 100% of endodontic treatments were performed using the manual technique. Two alternative scenarios were then proposed. The first replacing the manual technique with a rotary technique and the second, with a reciprocating technique. As the measure of effectiveness used in this study was the clinical time per procedure, we also considered this issue when calculating the number of possible procedures in the alternative scenarios. In this way, we predicted a demand that would make it possible to perform the quantities of treatments shown in Table 1.

Since the difference between the scenarios (reference and alternative) was in the number of treatments performed and in the values of these treatments, the incremental financial impact (IFI) was calculated? According to the formula expressed below:

$${\text{IFI}} = \left( {{\text{No}}_{{{\text{alternative}}}}^{{}} \, \times \,{\text{Cost}}_{{{\text{tradicional}}}} } \right) - \left( {{\text{No}}_{{{\text{alternative}}}}^{{}} \, \times \,{\text{Cost}}_{{{\text{tradicional}}}} } \right)$$

where IFI = Incremental Financial Impact. No = Total number of teeth treated.

Therefore, two IFIs were calculated. The IFI of the rotational technique (alternative 1) in comparison with the manual technique (traditional) and the IOI of the reciprocating technique (alternative 2) in comparison with the manual technique (traditional). The US dollar exchange rate was 1US= R$5.16.

## Results

The entire micro-costing technique was based on direct costs (Human resources, materials, instruments, equipment). The majority of the costs were found to be related to the human resources used (CD clinical hour + ASB) in the average value of R$ 96.67 (R$ 78.98 h for the CD and R$ 17.69 h for the ASB). While the costs of material, instruments and equipment ranged from the lowest value R$ 35.89 (manual technique for single-rooted technique) to the highest value R$ 106.58 (tri-root with reciprocating technique). To obtain the total value of the technologies tested, the following factors?/variables?/were taken into account: number of canals, average costs of materials, HR expenses, average time to perform endodontic treatments and the number of endodontic treatments performed in 2019 according to TABNET/DATASUS/Brazil. The cost data for each technique are presented in the Additional file1: Table S1 and are expressed in reais (year 2019).

Table 2 presents treatment costs, effectiveness and cost-effectiveness ratio (CER). The CER of each treatment corresponds to the division between its cost and its effectiveness. The incremental cost-effectiveness ratio (ICER) was division between the incremental cost and the incremental effectiveness of the alternative technology compared with the baseline strategy (manual technique). When compared with the manual technique, alternative techniques were cost-effective. In particular, the reciprocating technique in teeth with more than two roots.

**Table 2:** Individual cost, cost-effectiveness ratio of each technique and incremental cost-effectiveness ratio of alternative techniques in relation to the traditional technique

Number of dental canals	Technique	Cost^a^ per treatment in R$	Effectiveness (time in minutes)	RCE^b^ (R$/minute)	RCEI^c^ (R$/minute)
Single-rooted	Manual	133.12	20.00	6.66	Reference
	Rotary	135.73	21.95	6.18	1.34
	Reciprocal	128.28	19.77	6.49	21.04
Bi-radicular	Manual	138.61	53.88	2.57	Reference
	Rotary	155.93	25.15	6.20	− 0.60
	Reciprocal	154.61	32.05	4.82	− 0.73
Tri-radicular or more	Manual	165.01	60.30	2.74	Reference
	Rotary	163.48	45.33	3.61	0.10
	Reciprocal	191.44	50.88	3.76	− 2.81

^a^Direct cost of techniques/materials + CD and ASB clinical time

^b^RCE: Incremental cost-effectiveness ratio of each technology

^c^RCEI: Incremental cost-effectiveness ratio of the alternative technology in relation to the reference technology (manual technique)

By multiplying the number of endodontic treatments performed per year by the average cost invested per treatment, we arrive at an average annual investment value to guarantee care of the population.

The incremental financial impact of replacing manual endodontic treatments with rotary endodontic treatments would be − R$2060963.66 in the case of single-rooted teeth, but the number of treatments would also be reduced (− 19,379). In the case of two-rooted teeth, the incremental financial impact would be BRL 34921540.62 with the possibility of performing an additional 204,110 treatments. In turn, BRL 11523561.50 represented the incremental financial impact for teeth with three or more roots and with an increase of 72,545 procedures (Table 3).

**Table 3:** Analysis of the replacement of the manual instrumentation technique by the rotary or reciprocating technique

Number of dental canals	Technique	Number of endodontics per year	Cost of technology^a^	Annual investment	Difference in the number of endodontics per year	Annual financial impact
Single-rooted	Manual	218.141	133.12	R$ 29.038.92992	Reference	Reference
	Rotary	198.762	135.73	R$ 26.977.96626	− 19.379	− R$ 2.060.96366
	Reciprocal	220.679	128.28	R$ 28.308.70212	+ 2.538	− R$ 730.22780
Bi-radicular	Manual	178.676	138.61	R$ 24.766.28036	Reference	Reference
	Rotary	382.786	155.93	R$ 59.687.82098	+ 204.110	R$ 34.921.54062
	Reciprocal	300.376	154.61	R$ 46.441.13336	+ 121.700	R$ 21.674.85300
Tri-radicular or more	Manual	219.670	165.01	R$ 36.247.74670	Reference	Reference
	Rotary	292.215	163.48	R$ 47.771.30820	+ 72.545	R$ 11.523.56150
	Reciprocal	260.340	191.44	R$ 49.839.48960	+ 40.670	R$ 13.591.74290

^a^Average direct cost of techniques/materials + CD and ASB clinical hour

When we analyzed the incremental financial impact of replacing manual endodontic treatments with reciprocating endodontic treatments, it would be—R$ 730227.80 in the case of single-rooted teeth, allowing for an additional 2538 treatments. In turn, R$ 21674853.00 represented the incremental financial impact for bi-radicular teeth, with an increase of 121,700 procedures. In the case of two-rooted teeth, the incremental financial impact would be BRL 13591742.90 with the possibility of performing an additional 40,670 treatments (Table 3).

## Discussion

The time outcome considered in this study proved to be important, since DSCs end up failing to achieve their goals, especially in endodontic treatments. The minimum target can be considered too high. Some teeth, such as molars because they have 2 or 3 roots, require a longer time and more treatment sessions, depending on the case. Therefore, the demand for service accumulates and leads to a longer waiting times.

The main advantage of incorporating this new technology would be the number of endodontic treatments completed, and thus, the emergence of new opportunities for referral to DSCs, ensuring that the population would have greater access to this treatment. On the other hand, it should be noted that the greater number of treatments completed promoted a financial impact that must be borne by the payer, so that management would be willing to pay.

Single-session treatment was another advantage found using these new technologies. Instrumentation with the aid of devices allowed complete resolution of periapical radiolucent areas even in cases of periapical lesions from endodontic treatments in a single session. This allowed for a shorter time to complete treatments and thus a higher number of completed endodontic treatments [10].

However, this increase in the number of completed endodontic treatments would not only be able to exceed the goals stipulated for each type of DSC but would also extrapolate this minimum. In this context, the need for public policies to encourage not only the achievement of goals was reinforced, but, in cases of exceeding them, the municipalities could also receive more financial resources with a view to expanding the offer and reducing the waiting list and pent-up demand in the public sector. In addition, we can say that the reciprocating technique was shown to be a technology with a good cost-effectiveness ratio, and it could be self-funded by the municipalities through the transfer of federal incentives by the Ministry of Health (MS), which could positively impact the quality of life of the users the would be benefited.

The DSCs offer services in the specialties of endodontic treatments, Periodontics, Patients with Special Needs and Minor Oral Surgery, where they are classified into three types and receive monthly transfers from the MS for funding and implementation. The transfer for funding of DSC type I (three dental chairs) was R$ 8250.00 for funding; Type II DSC (with four or more seats), whose monthly amount received was R$ 11000.00, and Type III DSC (with at least seven seats), whose cost resource was R$ 19250.00. There are 853 units in operation distributed throughout the national territory and they were responsible for an increase from 6 million specialized procedures to 25 million procedures, between 2002 and 2010 [10].

The resources transferred by the MS to the DSCs can be used for the incorporation of technology, helping them to reach the goals, which in turn would mean that resources will not be cut. However, there is a need for new public policies to reformulate and update these values that have been assigned since the implementation of ordinance no 1.341 (06/2012). Therefore, in addition to meeting the goals, they would be able to exceed them by using this new technology.

Thus, the Ministry of Health has determined that at least 20% of dental procedures must be carried out in each area. Moreover, it is through the Outpatient Information System of the Unified Health System (SIA-SUS) program that the management forwards the data, and thus, it is possible to monitor each procedure performed [11]. Within this context, as regards caries, it still affects a large part of the population of many countries. A recent survey carried out in Brazil showed that on average 4 teeth were affected by caries in children between 6 and 12 years of age, of these, an average of 68.5% will need endodontic treatment [12].

Knowing that approximately 70% of the population depends on the SUS and that there is a process of migration of patients from private to public health plans, due to economic and political crises, it is important to make the health system more efficient in the sense of spending more wisely and maximizing health gains. Since resources tend to be scarce and demand tends to be growing, due to the economy itself, increasing numbers of users are moving from the private to the public sector [13].

The financing of the health sector in Brazil is shared by the Union, States and Municipalities, based on the legal framework of the Federal Constitution and Federal Laws 8080 and 8142 of 1990 [14], in addition to others that contributed to the regulation: Complementary Law No. 141 of January 13, 2012, which defined the types of actions and health services, Constitutional Amendment 95/2016 (law on expenditure ceiling) in which total public spending will be readjusted based on the official inflation of the previous year for a period of 20 years and, more recently, by Ordinance #3.992 [15].

Although there are studies available, so far few advances have been made with regard to the materials used since the main component of the files is stainless steel which, in spite of its elastic properties and resistance to fracture, has not proved to be totally suitable for conformation of the root canal system. The most obvious advantage, when the two procedures were compared, was the time savings, due to the possibility of working with rotary instrumentation at high rotations, more continuously and using fewer instruments [16–18].

In the manual endodontic system, the movements of rotation completely depend on the operator, who can choose the technique, while in the mechanized system, various types of movements can be selected depending on the specificity of the system chosen. In the present study, continuous movement was described, which, as its name implied, was movement that remained in the same, generally clockwise direction. While in reciprocating movement, which consists of a counterclockwise rotating movement that will allow the dentin to be cut, and then a shorter movement rotating in the clockwise direction to release the instrument [17].

At present rotary systems are independent of the manual system and each system has its advantages and limitations. However, it is clear that Reciproc® and WaveOne® single file reciprocating systems are safe to use, provided that the manufacturer's instructions are followed [19]. These systems rely on the use of a single file, thereby reducing the number of instruments to be used, especially for retreatment; that is, no additional instruments are required. Furthermore, if used according to the manufacturer’s instructions, there is a lower possibility of fractures, a decrease in the probability of cross-infection; and a lower learning curve for use of this type of instrumentation system.

The studies that presented the best evidence observed similarity between rotating and alternative systems in relation to bacterial disinfection and endotoxin reduction. However, they reinforced the need for further studies with the most recent instruments [20]. In a systematic review with meta-analysis of in vitro studies, comparing rotary and manual systems, they reported superior characteristics in rotary instrumentation, such as shorter instrumentation time, maintenance of centralization without canal deviation, and canal shaping. On the other hand, manual instrumentation obtained better results in relation to debris production, smear layer removal, lower production of dentinal defects and greater number of surfaces touched during instrumentation. Despite the large number of studies addressing the mechanical quality of instruments, no research was found that addressed the costs used in different technologies [21]. In another perspective [22], they evaluated the cost-effectiveness of endodontic treatment in one or several consultations from the perspective of the German health system and demonstrated the importance of this type of evaluation for the therapeutic decision.

The present study was developed in two phases, the first being a pilot study to survey the costs and clinical time of the procedures. In this phase, we sought to control the main bias related to the clinical differences between the techniques, that is, the experience of the professional who performs the care, due to the expertise in the three techniques tested. Since the factor most affects the cost of the technique is the time taken to perform it, and number of queries that arise. For this reason, the 3 techniques were performed by the same professional.

Because the study concerned a reference service (endodontic treatments in DSC's), the patient was referred to undergo only the specialized treatment (in this case, endodontic treatments) and would then be counter-referred to the basic unit of origin to conclude treatment with restoration of this same tooth and completion of the necessary dental treatment. The treatments were performed in a single session and positive results were reported, mainly with regard to postoperative pain and also with the purpose of finalizing referrals from the basic units and thus reducing the demand for the specialized service [23].

In other studies, different results were found, and the differences were not significant for the intervention. However, few economic evaluations have been developed with this particular objective, that is, to compare endodontic techniques. Some authors have found a shorter root canal instrumentation time with the use of rotary systems when compared with conventional systems [21, 24], while others have emphasized the single reciprocal instrument [25, 26], which could impact the routine of consultations at the clinic, in addition to providing both patients and professionals with greater comfort.

It is necessary to emphasize that endodontic therapy requires other phases in addition to instrumentation. Despite all the technological development in the sector, there is still no instrument that meets all the requirements for optimal root canal preparation [27]. This fact highlights the importance of choosing a good irrigation solution and a filling that promotes three-dimensional sealing of the root canals. Another important point to emphasize is that the three systems represented safe alternatives for root canal preparation, but no system has been shown to be an absolute substitute for manual instrumentation.

In addition to the characteristics already mentioned, it is essential for the manager to gather other information that can help with the decision to incorporate a new technology. As previously observed, the number of sessions has an important impact on the final cost and the agility acquired with the use of mechanized systems can contribute to performing treatment of both types of pathologies evaluated here, in a single session, corroborating the findings of Almeida et al. [23], who stated that in the dental clinic, biological criteria, professional skill, patient comfort and time optimization, in addition to financial resources, should guide the dentist's decision.

The results of this study reported that the savings from additional treatment performed by using the rotary technique generated a more cost-effective financial impact that could be financed by the transfers from the MS. Our study assumed the similarity between the chosen clinical outcome of the techniques used (success rate of endodontic therapy). The option for the strategy with the greatest impact on efficacy, quality of endodontic treatment and financial aspect, took into account the evidence found in the literature, but the public sector must be willing to pay for this new technology.

Efficiency, understood as being the relationship between results and the costs to obtain them, can be allocative, productive or technical. The first being defined as the ability to associate resources and results in optimal proportions, the second would be the ability to get everything one possibly can out of the scarce resources available. Finally, technical efficiency refers to the ability to produce a given level of production with a minimum amount of inputs, with the use of a given technology. In other words, there must be no waste of resources in the production process, and technical efficiency is the relationship between production observed and potential production. When one of the alternatives subject to analysis is the most effective and also the cheapest, the choice is clear. The difficulty arises when the most effective treatment is also the most expensive. If we use ICER as a single analysis of a procedure, it must be assumed that both alternatives are feasible and cost-effective.

The technique that made the best use of resources, demonstrated by the allocative efficiency, was the roundabout, where an incremental financial impact was found with the replacement of manual endodontic treatments by rotary, mainly in cases of bi-radicular teeth, of R$ 34921540.62 with the possibility of performing an additional 204,110 treatments and an incremental financial impact of R$ 11523561.50 for teeth with three or more roots and with an increase of 72,545 procedures. The most efficient procedure in terms of production were in two-rooted teeth with use of the mechanized techniques, allowing the accomplishment of an additional 204,110 treatments by the rotary and 121,700 treatments by the reciprocating systems.

In technical terms, using the smallest amount of money to meet the existing demand for endodontic treatment, would promote an annual financial impact of − R$ 2060963.66 for treatment of single-rooted teeth by replacing the manual technique with the reciprocating technique, and − R$ 730227 0.80 for single-rooted teeth using the reciprocating technique.

When we consider the financial impact of implementing these new technologies and the number of endodontic treatments that could be performed by incorporating them, we have the incremental cost-effectiveness ratio (ICER). The amount required for additional treatment performed using the rotary technique was R$1.34 for single-rooted teeth, -R$0.60 for two-rooted teeth and R$0.10 for tri-rooted teeth or more. For the reciprocating technique, it cost BRL 21.04 for single root,—BRL 0.73 for two root and—BRL 2.81 for three or more roots. Thereby, the replacement of manual technology, which is currently the reference in most DSC's, with mechanized technologies was observed to become feasible, even if initially performed in single and bi-rooted teeth and later in teeth with three or more roots. Because this replacement resulted in an increase in the number of cases completed and thus, a decrease in the waiting list. When the efficiency between the two mechanized techniques was compared, the reciprocating technique was outstanding for the higher number of treatments completed in the same time interval.

Finally, the main finding of this study was that the reciprocating instrumentation technique in endodontic treatments proved to be more cost-effective, since it showed treatment in a shorter clinical time and, therefore, a decrease in the costs associated [28, 29]. The costs of incorporation of this technology (reciprocal) could be paid by the municipalities through transfers of federal incentives from the Ministry of Health. Analysis of the composition of the direct costs of each technique demonstrated the importance of each item in the formation of the final cost. Despite the importance of the initial cost of implementing rotary and reciprocating technology, with the purchase of the equipment and instruments necessary to carry out each of them, the investment applied will be converted into endodontic treatments performed, thus reducing the demand for this treatment.

## Conclusion

The reciprocating technique could improve access to endodontic treatment in the SUS as it simultaneously allowed a reduction in clinical time and in the costs associated with consumables, patient displacement and/or absences from work to complete the treatment.. In addition, the completion of endodontic treatment in a shorter clinical time led to optimization of human resources, allowing the expansion of access to treatment by users. In addition, this technique could be self-funded by the municipalities through transfers of federal incentives by the Ministry of Health. However, it is worth noting that in the period of 1 year evaluated, the higher number of endodontic treatments performed promoted a financial impact that would have to be absorbed by the payer, so that it would be necessary for management to be willing to pay the amount of this impact.

## Supplementary Information

**Additional file1**. **Table S1**: Microcosting of the analyzed endodontic instrumentation techniques.

## Data Availability

All data generated or analyzed during this study are included in this published article [and its supplementary information files].
