# Supplementary material for: Economic analysis of the different endodontic instrumentation techniques used in the Unified Health System
Source: BMC Oral Health. 2022 Aug 11;22:344. doi: 10.1186/s12903-022-02369-x (PMC9373286; doi:10.1186/s12903-022-02369-x)
Supplement: Supplementary file 1 — Additional file1. Table S1: Microcosting of the analyzed endodontic instrumentation techniques. [file 12903_2022_2369_MOESM1_ESM.docx]

| **Table 4 (S1). Microcosting of the analyzed endodontic instrumentation techniques.** | | | | |
| --- | --- | --- | --- | --- |
| ***Common resources to the three techniques*** | | ***Custo médio por procedimento*** | | |
|  |  | ***Single-rooted teeth*** | ***Bi-radicular teeth*** | ***Tri-radicular teeth or more*** |
| ***Human resources*** | |  |  |  |
|  | Dentist | 78,98 | 78,98 | 78,98 |
|  | Oral Health Assistant | 17,69 | 17,69 | 17,69 |
| ***Consumables*** | |  |  |  |
|  | 70º ethyl alcohol | 0,0051 | 0,0051 | 0,0051 |
|  | Apron /coat | 9,0300 | 9,0300 | 9,0300 |
|  | Procedure glove | 0,2300 | 0,2300 | 0,2300 |
|  | Cap | 0,0400 | 0,0400 | 0,0400 |
|  | Surgical mask | 0,2200 | 0,2200 | 0,2200 |
|  | N95 mask | 4,8300 | 4,8300 | 4,8300 |
|  | Sugador descartável | 0,2900 | 0,2900 | 0,2900 |
|  | Sterile protection for the body | 4,8000 | 4,8000 | 4,8000 |
|  | Needle for cartridge | 0,2600 | 0,2600 | 0,2600 |
|  | Anesthetic | 0,8100 | 0,8100 | 0,8100 |
|  | Gauze compress | 0,2100 | 0,2100 | 0,2100 |
|  | Cotton roll | 0,0600 | 0,0600 | 0,0600 |
|  | Eugenol | 0,0272 | 0,0272 | 0,0272 |
|  | Formocresol | 0,0295 | 0,0295 | 0,0295 |
|  | Rubber dam | 0,5800 | 0,5800 | 0,5800 |
|  | Syringe | 0,3400 | 0,3400 | 0,3400 |
|  | Chlorhexidine Gel | 0,4276 | 0,4276 | 0,4276 |
|  | Dakin liquid | 0,0610 | 0,0610 | 0,0610 |
|  | Saline | 0,0051 | 0,0051 | 0,0051 |
|  | Irrigation needle | 0,0300 | 0,0300 | 0,0300 |
|  | Aspiration cannula | 0,8300 | 0,8300 | 0,8300 |
|  | Absorbent Paper Cone | 0,1400 | 0,1400 | 0,1400 |
|  | Guta Percha Cone | 0,1200 | 0,1200 | 0,1200 |
|  | Scalpel blade | 0,2500 | 0,2500 | 0,2500 |
|  | Obturator cement - Powder | 0,2178 | 0,2178 | 0,2178 |
|  | Obturator cement - Liquid | 0,0976 | 0,0976 | 0,0976 |
|  | Ethyl alcohol 92.8º | 0,0100 | 0,0100 | 0,0100 |
|  | CIV Powder | 0,3320 | 0,3320 | 0,3320 |
|  | CIV Líquid | 0,0109 | 0,0109 | 0,0109 |
|  | Coltosol | 0,2146 | 0,2146 | 0,2146 |
|  | Palito de Fósforo | 0,0200 | 0,0200 | 0,0200 |
| ***Permanent material*** | |  |  |  |
|  | Protective glasses | 0,0007 | 0,0007 | 0,0007 |
|  | Face Shield | 0,0022 | 0,0022 | 0,0022 |
|  | Clinical mirror | 0,0004 | 0,0004 | 0,0004 |
|  | Clínical probe nº 47 | 0,0006 | 0,0006 | 0,0006 |
|  | Clinical clamp 13 cm | 0,0009 | 0,0009 | 0,0009 |
|  | sculptor Hollemback nº 03 | 0,0008 | 0,0008 | 0,0008 |
|  | Dentin Digger nº17 | 0,0008 | 0,0008 | 0,0008 |
|  | Condenser nº1 | 0,0023 | 0,0023 | 0,0023 |
|  | Condenser nº3 | 0,0023 | 0,0023 | 0,0023 |
|  | Syringe Carpule | 0,0028 | 0,0028 | 0,0028 |
|  | Broca esférica | 0,0002 | 0,0002 | 0,0002 |
|  | High, low, contra-angle academic kit | 0,0552 | 0,0552 | 0,0552 |
|  | Ostby Arch | 0,0008 | 0,0008 | 0,0008 |
|  | rubber sheet punch | 0,0090 | 0,0090 | 0,0090 |
|  | Clip Holder | 0,0055 | 0,0055 | 0,0055 |
|  | Clip | 0,0011 | 0,0011 | 0,0011 |
|  | Tamborel | 0,0013 | 0,0013 | 0,0013 |
|  | Mechanical instrumentation engine + Apex Locator | 0,8737 | 0,8737 | 0,8737 |
|  | Gates drill | 0,0009 | 0,0009 | 0,0009 |
|  | Largo drill | 0,0008 | 0,0008 | 0,0008 |
|  | Cuba de inox | 0,0020 | 0,0020 | 0,0020 |
|  | Stainless steel tubor metálico + ponta sugador | 0,0053 | 0,0053 | 0,0053 |
|  | Millimeter ruler | 0,0036 | 0,0036 | 0,0036 |
|  | Scalpel blade º 15 | 0,0248 | 0,0248 | 0,0248 |
|  | Glass plate | 0,0005 | 0,0005 | 0,0005 |
|  | Spatula nº24 | 0,0010 | 0,0010 | 0,0010 |
|  | Alcohol lamp | 0,0035 | 0,0035 | 0,0035 |
| ***Total*** | | ***122,2014*** | ***122,2014*** | ***122,2014*** |
| ***Specific resources for each technique*** | | ***cost per service*** | | |
|  |  | ***Single-rooted teeth*** | ***Bi-radicular teeth*** | ***Tri-radicular or more*** |
| Lima manual | | 1,6633 | 1,6633 | 1,6633 |
| Average manual technical cost | | 133,12 | 138,61 | 165,01 |
|  | |  |  |  |
| Rotary lime | | 9,5766 | 2,3034 | 11,8800 |
| Average technical cost of rotation | | 135,73 | 155,93 | 163,48 |
|  | |  |  |  |
| Reciprocating lime | | 20,695 | 12,935 | 33,6300 |
| Reciproc Calibrated Gutta Cone | | 0,8600 | 0,8600 | 0,8600 |
| Average technical cost of Reciprocating technical | | 128,28 | 154,61 | 191,44 |
